# Thermoresponsive Shape-Memory Biobased Photopolymers of Tetrahydrofurfuryl Acrylate and Tridecyl Methacrylate

**DOI:** 10.3390/ma16062156

**Published:** 2023-03-07

**Authors:** Justinas Jaras, Aukse Navaruckiene, Jolita Ostrauskaite

**Affiliations:** Department of Polymer Chemistry and Technology, Kaunas University of Technology, Radvilenu Rd. 19, LT-50254 Kaunas, Lithuania

**Keywords:** biobased photopolymers, photocuring, shape-memory, thermo-responsive

## Abstract

A series of thermoresponsive shape-memory photopolymers have been synthesized from the mixtures of two biobased monomers, tetrahydrofurfuryl acrylate and tridecyl methacrylate, with the addition of a small amount of 1,3-benzendithiol (molar ratio of monomers 0–10:0.5:0.03, respectively). Ethyl (2,4,6 trimethylbenzoyl) phenylphosphinate was used as photoinitiator. The calculated biorenewable carbon content of these photopolymers was in the range of (63.7–74.9)%. The increase in tetrahydrofurfuryl acrylate content in the photocurable resins resulted in a higher rate of photocuring, increased rigidity, as well as mechanical and thermal characteristics of the obtained polymers. All photopolymer samples showed thermoresponsive shape-memory behavior when reaching their glass transition temperature. The developed biobased photopolymers can replace petroleum-derived thermoresponsive shape-memory polymer analogues in a wide range of applications.

## 1. Introduction

Taking into account recent environmental problems, the development of new sustainable polymers by green chemistry and green engineering concepts has attracted a great deal of attention from scientists and industry. Photopolymerization, an environmentally friendly method of polymer production, is a fast and easily controlled process. Moreover, it is possible to cure selected areas of resin and perform it at room temperature or even lower temperatures, which simplifies the polymer product manufacturing [1,2]. Biobased photopolymers have great potential to replace petroleum-based polymers not only in conventional areas such as films, coatings, inks, and adhesives, but also in high-tech areas such as microelectronics, optoelectronics, nanotechnology, and others [3,4,5]. Recently, a wide range of novel photocurable monomers have been developed from renewable resources for the production of high performance photopolymers [6]. However, their thermal, mechanical, and other properties still need to be optimized to compete with the well-studied and wildly used petroleum-based polymers.

In this work, two biobased monomers, tetrahydrofurfuryl acrylate (THFA) and tridecyl methacrylate (C13-MA), have been selected for the synthesis of photopolymers. C13-MA is derived from natural oils, and its long alkyl chains can provide flexibility for synthesized polymers [7]. However, only a couple of papers have been published on photopolymers with C13-MA fragments, a promising monomer but still little studied [7,8]. Other examples of highly promising biobased monomers are tetrahydrofurfuryl (meth)acrylates derived from hemicellulose [8,9]. Sustainable elastomers with a semi-interpenetrating network and with shape-memory and self-healing properties were prepared using tetrahydrofurfuryl methacrylate (THFMA) [10]. Polymers with THFMA fragments have been used in stereolithography [11], drug delivery [12], and dental applications [13]. In radical polymerization, acrylates are more reactive than methacrylates, as secondary acrylate radicals are very unstable compared to highly stable tertiary methacrylate radicals [14]. However, only the use of tetrahydrofurfuryl acrylate (THFA) in the production of organic coatings has been published to date [9].

Stimuli-responsive materials are known as smart materials because they are aware of environmental stimuli and can give straight responses [15]. Many biobased polymers with great shape-memory properties were obtained [16,17]. However, the synthesis of new biobased shape-memory polymers by environmentally friendly photocuring has not yet drawn much scientific attention [18]. Therefore, the aim of this work was to develop new biobased thermoresponsive shape-memory polymers by photocuring using C13-MA and THFA, which are still poorly investigated. Only a few articles are published on thermoresponsive shape-memory biobased photopolymers, showing that it is a new and promising type of material [19,20,21].

In this study, THFA and C13-MA were selected as main components to find the best composition of photopolymers with thermo-responsive shape-memory properties. Photoinitiator ethyl (2,4,6-trimethylbenzoyl) phenylphosphinate was used to assure the high absorption wavelength [22] and thus high reaction rate [23]. Organic solvents, which are often environmentally damaging components in chemistry, were not used in the preparation of photopolymer samples [24]. The increase in THFA content in the photocurable resins resulted in a higher rate of photocuring and improved mechanical and thermal properties. A small amount of dithiol was added to ensure shape-memory properties, increase flexibility, and reduce the brittleness of polymers, as the increase in the amount of THFA made them more rigid but brittle. A series of compositions were developed to find the best mixture and determine the point at which the increase in THFA is no longer useful. The developed biobased photopolymers can replace petroleum-derived thermoresponsive shape-memory polymer analogues in a wide range of applications.

## 2. Materials and Methods

### 2.1. Materials

**Figure 1:**
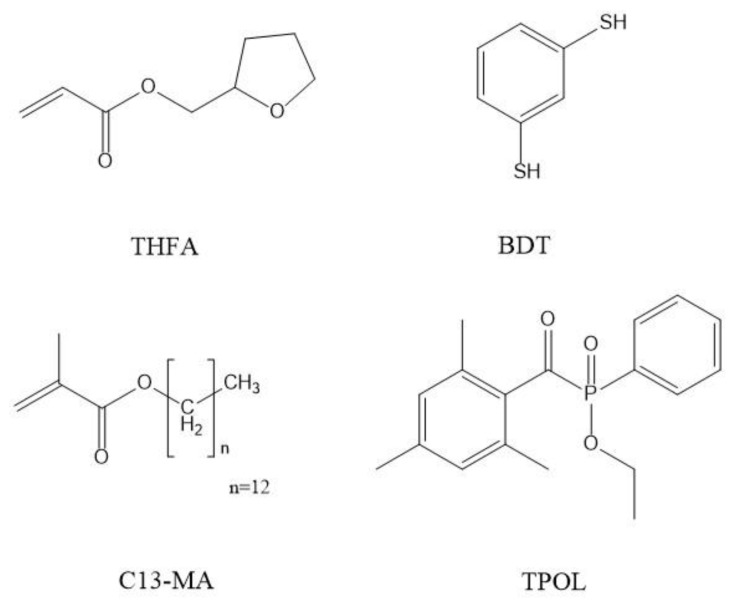
Chemical structures of tetrahydrofurfuryl acrylate (THFA), 1,3-benzenedithiol (BDT), tridecyl methacrylate (C13-MA), and ethyl(2,4,6-trimethylbenzoyl) phenylphosphinate (TPOL).

Tetrahydrofurfuryl acrylate (THFA, SA5100 Arkema, Sartomer, Mulhouse, France), tridecyl methacrylate (C13-MA, VISIOMER^®^ Terra C13-MA, Evonik, Essen, Germany), 1,3-benzenedithiol (BDT, Fluorochem, Glossop, United Kingdom), and ethyl(2,4,6 trimethylbenzoyl) phenylphosphinate (TPOL, Fluorochem, Glossop, UK) (Figure 1) were used as received.

### 2.2. Preparation of Photopolymer Specimens

The mixtures containing 0 to 10 mol of THFA, 0.5 mol of C13-MA, 0.03 mol of BDT, and 3 mol.% of TPOL (Table 1) were stirred with a magnetic stirrer at room temperature (25 °C) for 5 min. When homogenous mixtures were obtained, the resins were poured into a Teflon mold and cured for 2–3 min under a UV/Vis lamp (Helios Italquartz, model GR.E 500 W, Milan, Italy), with UV/Vis light at an irradiance intensity of 310 mW/cm^2^.

**Figure 2:**
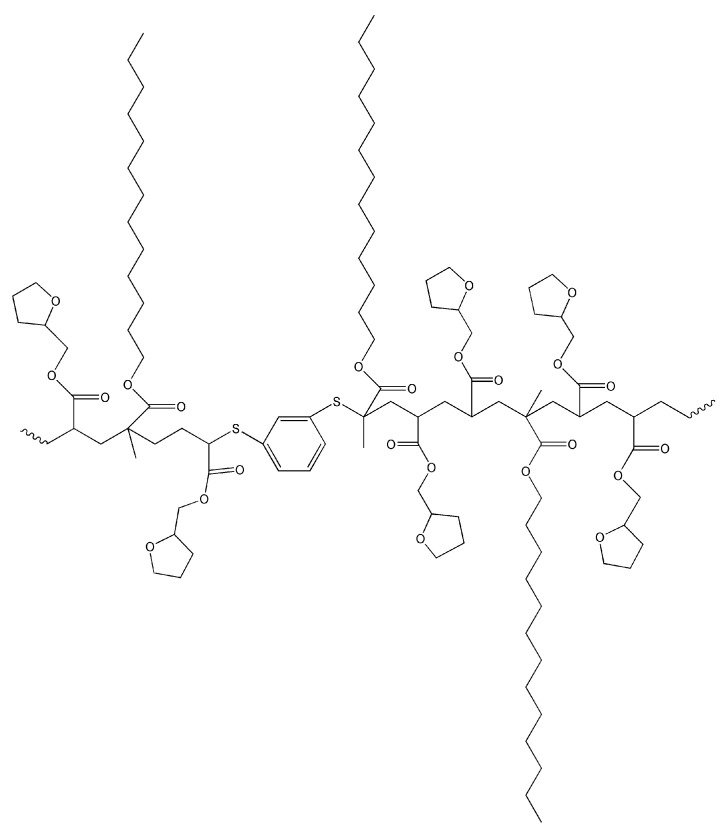
The possible polymer structure.

During the photocuring, the radical and thiol-ene photopolymerizations are taking place, and the random structure photopolymer is formed. The scheme of the possible polymer structure is presented in Figure 2.

The calculated values of the biorenewable carbon (BRC) content of the photopolymers ranged from 63.7% (**C10**) to 74.9% (**C0**).

### 2.3. Characterization Techniques

Fourier transformation infrared (FT-IR) spectra were recorded using a Spectrum BX II FT-IR spectrometer (Perkin Elmer, Llantrisant, UK). Reflection was measured during the test. The range of wavenumbers was (650–4000) cm^−1^.

The Soxhlet extraction was used to determine the yield of the insoluble fraction. Photopolymer samples of 0.2 g were extracted with acetone for 24 h. The insoluble fractions were then dried under vacuum until no changes in weight were observed. The yield of insoluble fraction was calculated as the difference of weight before and after extraction and drying.

The swelling values of the photopolymer samples were obtained by measuring the mass of the samples swollen in acetone and toluene at room temperature (25 °C). The initial mass of the polymer samples was measured before placing them into the solvent. The change in the mass of the samples was measured every 5 min until no change was observed. The swelling values were calculated according to the following equation:

(1) $$\alpha =\frac{M-M_{0}}{M_{0}}\cdot 100$$

where α is the swelling value (%); *M* is the mass of the swollen sample (g); *M*_0_ is the initial mass of the sample (g).

Thermogravimetrical analysis (TGA) was performed on a TGA 4000 apparatus (Perkin Elmer, Llantrisant, UK). A heating rate of 20 °C/min under nitrogen atmosphere (100 mL/min) was chosen. The temperature range of (10−600) °C was used. Aluminium oxide pans were used.

Dynamical mechanical thermal analysis (DMTA) was performed on an MCR302 rheometer (Anton Paar, Graz, Austria). The Peltier-controlled temperature chamber was used. The temperature was increased from −70 °C to 20 °C. The normal force was set at −0.1 N during the measurement. In all cases, the shear mode was used with a frequency of 1 Hz and a shear strain of 0.1%. The storage modulus (G′), the loss modulus (G″), and the loss factor (tanδ = G″/G′) were recorded as the functions of temperature. The glass transition temperature (*T*_g_) was determined as the maximum peak of the loss factor curve.

The mechanical properties of the synthesized polymers were determined by tensile and compression tests performed on a Testometric M500-50CT test machine (Testometric Co Ltd., Rochdale, UK) at room temperature (23 °C). The dimensions of the test specimens were 70 (±0.00) × 10 (±0.01) × 2 (±0.15) mm for the tensile test. The gap between the grips was set at 20 mm, and the test was performed at a speed of 5 mm/min until the sample broke. Young’s modulus, tensile strength, and elongation at break were determined. Five polymer samples were used to obtain the mean value and standard deviation.

The compression test was carried out using a selected force cell of 5000 N. The test specimens were subjected to a compression rate of 5 mm/min. Test specimens of 3 (±0.5) mm thickness and 15 (±0.05) mm diameter were used. All specimens were tested on CPR150 compression trays. The compression modulus (MPa) and the force required to fully crush the specimen (N) were determined. Five polymer samples were used to obtain the mean value and standard deviation.

The biorenewable carbon (BRC, %) content was calculated according to the following equation:

(2) $$\mathrm{BRC}=\frac{\mathrm{Bio}\;\mathrm{Sourced}\;\mathrm{Carbon}}{\mathrm{Bio}\;\mathrm{Sourced}\;\mathrm{Carbon}+\mathrm{Fossil}\;\mathrm{Carbon}}\;\cdot 100.$$

The shape-memory properties of the polymer samples were investigated using the bending technique. Specimens were mechanically bent and cooled below their glass transition temperature *T_g_*. Then, mechanical fixation was released, and the angle of shape fixity (*θ_f_*) was measured. The bent samples were heated above their glass transition temperature *T_g_* to induce return to permanent shape, and the angle of shape recovery (*θ_r_*) was measured. The shape fixity ratio (*R_f_*) and shape recovery ratio (*R_r_*) can be estimated according to Equations (3) and (4).

(3) $$R_{f}=\frac{180^{{}^{\circ}}-\theta_{f}}{180^{{}^{\circ}}}\times 100.$$

(4) $$R_{r}=\frac{\theta_{r}-\theta_{f}}{180^{{}^{\circ}}-\theta_{f}}\times 100.$$

*θ_f_* and *θ_r_* are the angles measured in the temporary shape and the permanent shape, respectively [25,26].

### 2.4. Real-Time Photorheometry

UV/Vis curing tests were performed with resins containing 0–10 mol of THFA, 0.5 mol of C13-MA, 0.03 mol of BDT, and 3 mol.% of TPOL on a MCR302 rheometer (Anton Paar, Graz, Austria) equipped with the plate/plate measuring system. The measuring gap was set to 0.1 mm, and the samples were irradiated with UV/Vis light in a wavelength range of 250–450 nm through the glass plate using the OmniCure S2000 UV/Vis spot curing system (Lumen Dynamics Group Inc., Mississauga, ON, Canada). The temperature was 25 °C. The shear mode with a frequency of 10 Hz and a shear strain of 1% was used in all cases. The storage modulus (*G*′), the loss modulus (*G*″), the loss factor (tan *δ*), and the complex viscosity (η*) were recorded as a function of the irradiation time. The values of each parameter were taken after 300 s of photocuring (Table 2). The gel point (*t_gel_*) is the intersection point of the G′ and G″ curves and was calculated from the onset of UV/Vis irradiation. The induction period was determined at the start of the increase in the *G*′ curve. The shrinkage was determined as the reduction in the height of the sample during the photocuring process. The normal force was set to 0 N during the measurement of the sample shrinkage.

## 3. Results

### 3.1. Monitoring of Photocuring Kinetics by Real-Time Photorheometry

**Figure 3:**
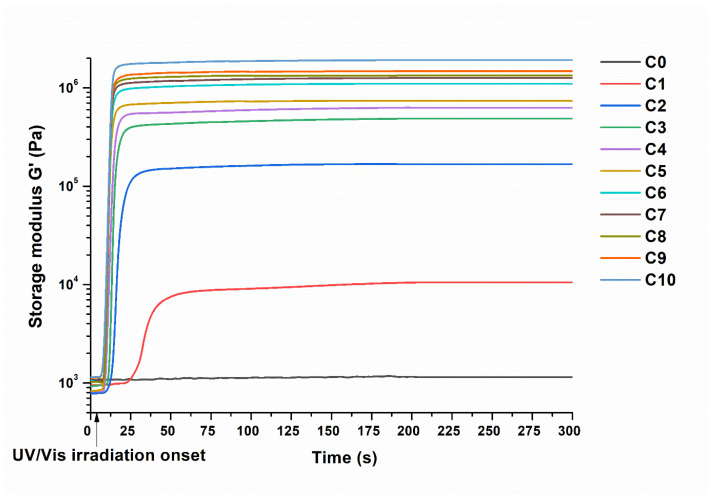
Dependency of the storage modulus G′ of resins **C0**–**C10** on the irradiation time.

The photocuring kinetics of the biobased resins was studied by real-time photorheometry. The dependencies of the storage modulus *G*′ on the irradiation time of the resins **C0**–**C10** are presented in Figure 3, and the rheological characteristics are summarized in Table 2. The change in the THFA content in the resins had a great influence on the photocuring kinetics. The resin **C0**, prepared without THFA, showed low values of *G*′ (0.001 MPa), and no gel point could be determined. The reason for this was the formation of a very soft polymer network from C13-MA having long alkyl chains [27] and BDT, which formed the flexible thioether linkages [28]. The THFA content was increased in the resins to find its optimum amount to obtain polymers with the highest rigidity while avoiding brittleness. The value of *G*′ was consistently increased from 0.011 to 1.915 MPa by increasing the amount of THFA from 1 mol to 10 mol in the resins. However, resins **C1**–**C3** showed higher values of *G*″ compared to *G*′, indicating that the viscous properties of the material dominated the elastic properties [29], and therefore, the polymers were viscous liquids. The **C4**–**C10** resins showed a greater value of *G*′ than *G*″, indicating that the samples went from viscous liquid to solid material [29]. The increasing amount of THFA resulted in the increase in the values of *G*′, which indicated the higher rigidity of the polymers.

The photocuring rate, described by the induction period and the gel point (*t*_gel_), is very important for the application of resins. The values of *t*_gel_ could not be determined for the **C0**–**C3** resins as their *G*″ was higher than *G*′. This showed that the resulting polymers were in a viscous liquid state [30]. The *t*_gel_ values of the resins **C4**–**C10** were the same for all resins (2.4 s), with the exception of the resin **C4** (*t*_gel_ = 9.6 s). The reason for this was the dominance of the influence of C13-MA and BDT components in the resin **C4** compared to other resins **C5**–**C10** containing a higher amount of THFA. For resins **C1**–**C3**, the induction period reduced from 19.2 to 2.4 s, with the increase in the amount of THFA from 1 mol to 3 mol in the resins. However, the induction period was constant when the amount of THFA increased from 3 to 10 mol. Therefore, the highest amount of THFA, which can affect the photopolymerization rate (t_gel_ and induction period) of the resins, is 5 mol. The shrinkage of the resins was highly dependent on the amount of THFA. It gradually increased from 1.0 to 8.1%, with an increase in the THFA content from 3 mol to 10 mol in the resins.

### 3.2. Characterization of Photopolymer Structure

**Figure 4:**
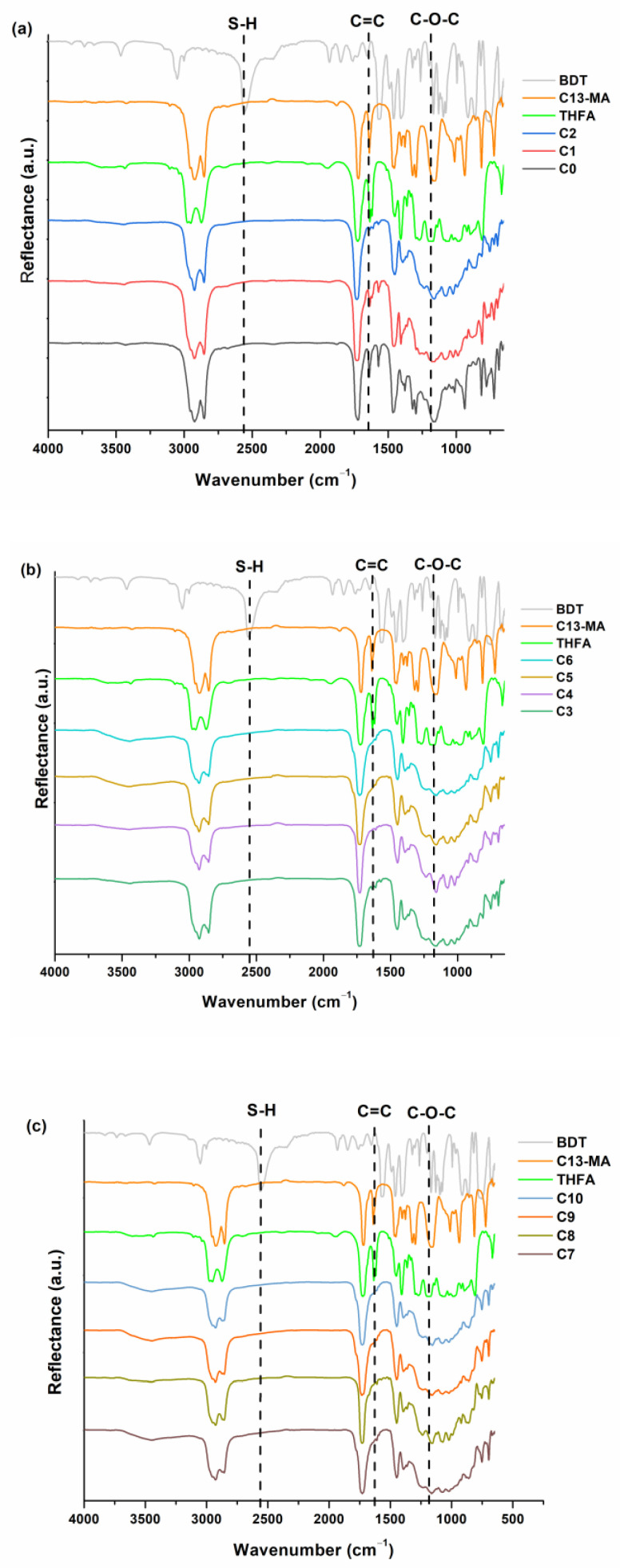
FT-IR spectra of THFM, C-13MA, BDT, and photopolymers **C0**–**C2** (**a**), **C3**–**C6** (**b**), **C7**–**C10** (**c**).

The chemical structure of the photopolymers was confirmed by FT-IR spectroscopy. The signals of the S-H group, which were present at 2560 cm^−1^ in the BDT spectrum, were not visible in the polymer spectra, confirming that all of these groups reacted during photocuring. The signals of the C=C group, which were present at 1606–1639 cm^−1^ in the C13-MA and THFA spectra, reduced or disappeared in the polymer spectra. The reduced signal of the C=C group was visible in the spectra of polymers **C0** and **C1**, indicating that not all these groups participated in the polymerization of these samples. The increased and overlapping signals in the range of ~800–1300 cm^−1^ in the polymer spectra indicated the formation of new S-C and C-C bonds in the polymers. FT-IR spectra of THFA, C13-MA, BDT, and polymers are presented in Figure 4.

Polymer samples **C0**–**C2** were not further studied as they were too soft.

The yield of the insoluble fraction of photopolymers **C3**–**C10** was in the range of 55–83% (Table 3) and increased with increasing THFA content up to sample **C9** (83%), while a slightly lower value was determined for the polymer **C10** (82%). Swelling values decreased with the increase in the THFA content up to sample **C9** from 168% to 119% in acetone, and from 296% to 139% in toluene due to the increased yield of the insoluble fraction, and the reduced cavities between macromolecules filled with an increasing amount of THFA fragments. The slightly higher swelling values determined for the polymer **C10** (120% in acetone and 142% in toluene) could be due to the lower yield of the insoluble fraction of this polymer. This was confirmed by the fact that the polymer samples with the higher amount of THFA (>10 mol) were brittle and not suitable for testing. The type of solvent also affected the swelling of the polymers. Polymers showed the higher swelling value in toluene than in acetone because the structure of the synthesized polymers was more similar to that of the nonpolar solvent toluene than to the polar solvent acetone [31].

### 3.3. Thermal Properties of Photopolymers

**Figure 5:**
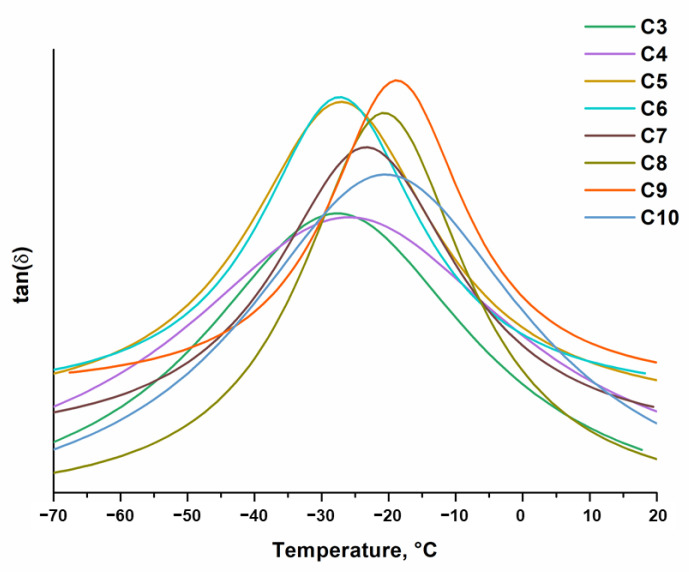
DMTA thermograms of photopolymers **C3**–**C10**.

DMTA and TGA were used to study the thermal properties of the polymers. Thermal characteristics are summarized in Table 3. The glass transition temperature (*T*_g_) of the photopolymers, determined from the DMTA curves (Figure 5), was in the range of −29–−19 °C. Such low *T*_g_ values can be explained by the presence of long and flexible alkyl chains in polymers, even if these polymers were solid materials at room temperature [8,9]. The *T*_g_ values of the polymers **C3**–**C10** were correlated with their yield of the insoluble fraction. It increased as the yield of the insoluble fraction increased.

**Figure 6:**
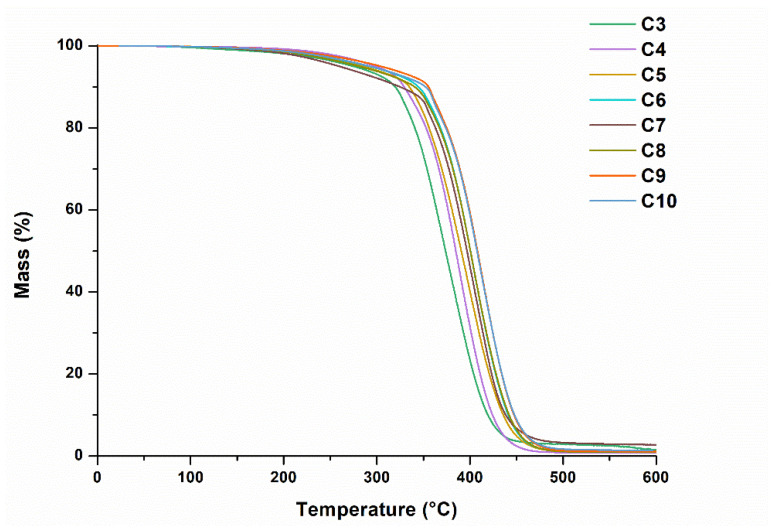
Thermogravimetric curves of photopolymers **C3**–**C10**.

The TGA revealed that the thermal decomposition of the photopolymers occurred In one step (Figure 6). The temperature of 15% weight loss (*T*_dec.-15%_) was in the range of 331–365 °C. The increased amount of THFA in compositions resulted in a higher thermal stability of the polymers. The **C3**–**C10** polymers synthesized in this work showed very high thermal stability compared to other biobased polymers [32].

### 3.4. Shape-Memory Properties of Photopolymers

**Figure 7:**
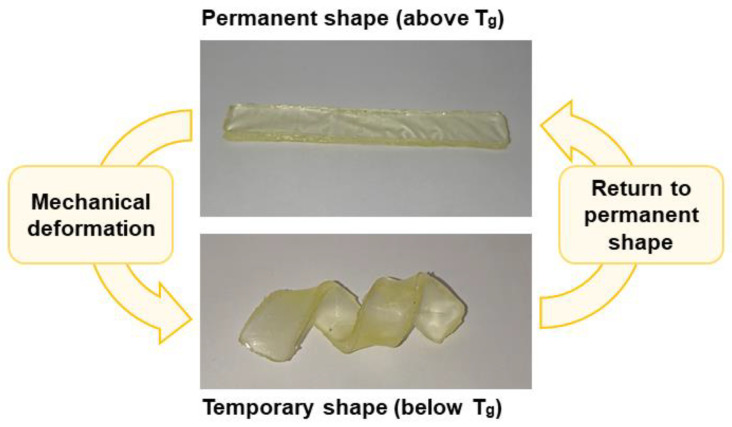
Shape-memory properties of polymer samples.

Thermoresponsive shape-memory properties of photopolymer samples were investigated by heating them above their *T*_g_, deforming them to a temporary shape, and cooling them down to a temperature below their *T*_g_ to fix the temporary shape. At this temperature, all of the polymer samples were hard and rigid materials that were able to sustain their temporary shape. To return to their permanent shape, the polymer samples were heated again above their *T*_g_, and all the polymer samples were able to return to their permanent shape within seconds (Figure 7).

The photopolymers developed in this work are composed of interlocked highly branched macromolecules with hard (benzene ring) and soft (aliphatic chains) fragments and with bulky substituents. For this reason, the molecular mobility of these macromolecules is hindered by strong physical bindings, which act as net points. The long aliphatic chains of synthesized polymers act as switchable units.

The shape-memory properties of the polymer samples were evaluated by the responses to the bending of the sample to determine the shape fixity ratio (*R_f_*) and the shape recovery ratio (*R_r_*). After measuring *θ_f_* and *θ_r_* angles, it was calculated that the shape fixity ratio (R_f_) in the temporary shape and the shape recovery ratio (*R_r_*) in the permanent shape, to which the sample returned after 1 s (at 50 °C), were 95% and 100%, respectively.

### 3.5. Mechanical Characteristics of Photopolymers

**Figure 8:**
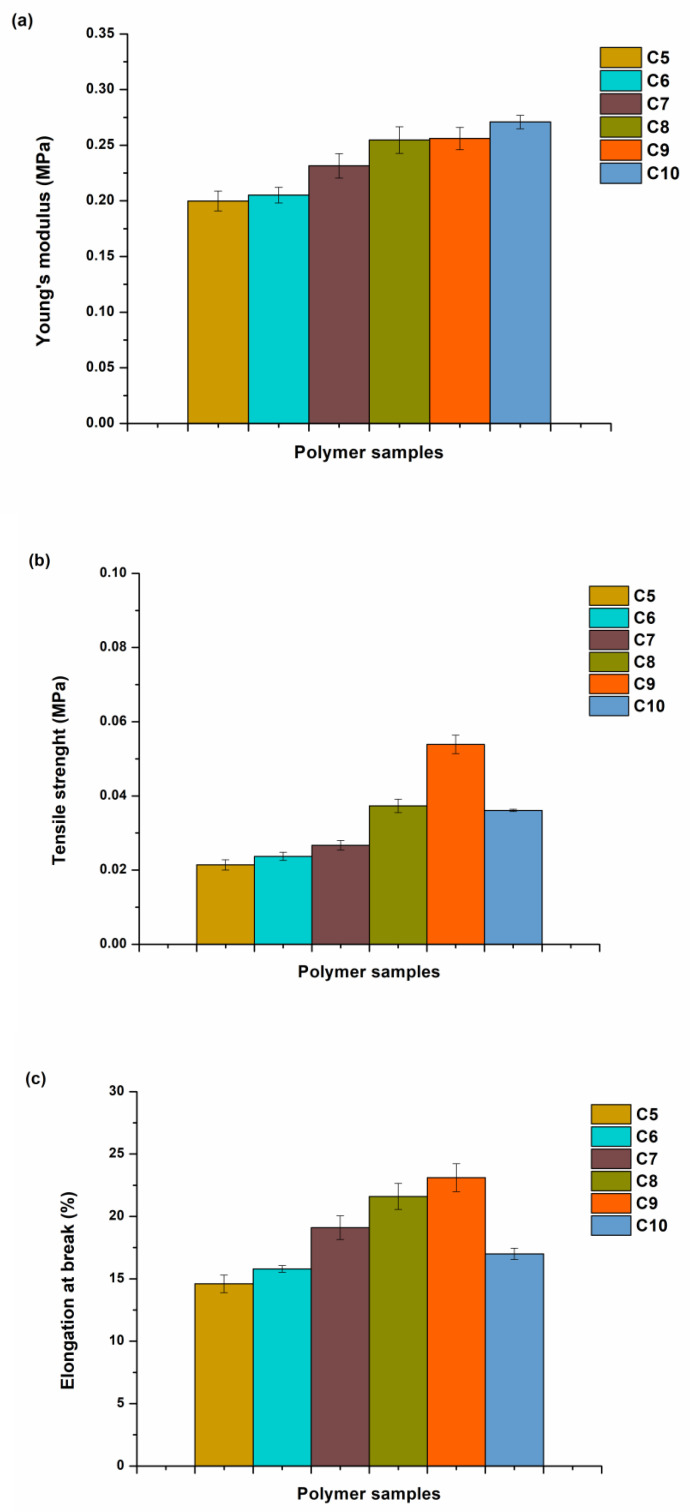
Tensile characteristics of polymers **C5**–**C10**. (**a**) Young’s modulus, (**b**) tensile strenght, (**c**) elongation at break.

**Figure 9:**
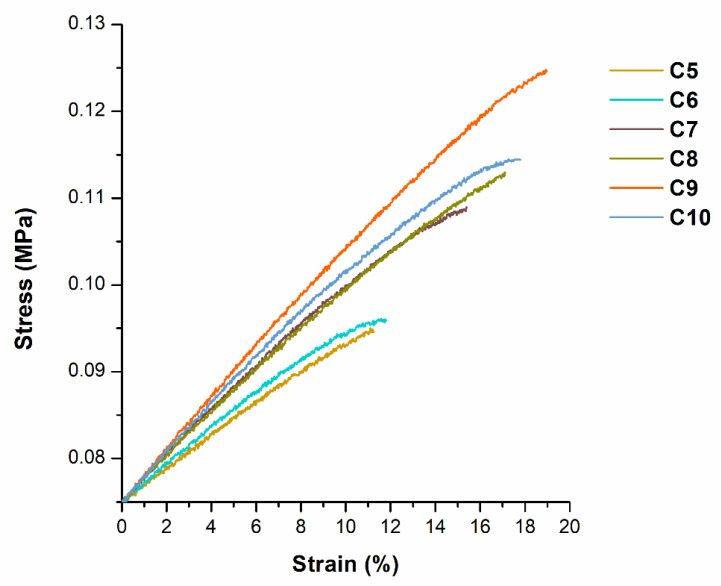
Tensile stress-strain curves of polymers **C5**–**C10**.

Tensile and compression tests were conducted to investigate the mechanical properties of the synthesized photopolymers. Polymer samples **C3**–**C4** were not tested as they were too soft. Young’s modulus values increased from 0.2 to 0.27 MPa, going from the polymer sample **C5** to **C10**, showing the impact of the higher content of THFA on this parameter (Figure 8 and Figure 9). However, the values of tensile strength (0.02–0.05 MPa) and elongation at break (14.61–23.15%) increased, only going from the polymer sample **C5** to **C9**. The lower values of the tensile strength and the elongation at break of polymer **C10** (0.04 MPa, 16.97%) than those of polymer **C9** (0.05 MPa, 23.15%) confirmed that too high amount of THFA led to increased brittleness of the polymer.

**Figure 10:**
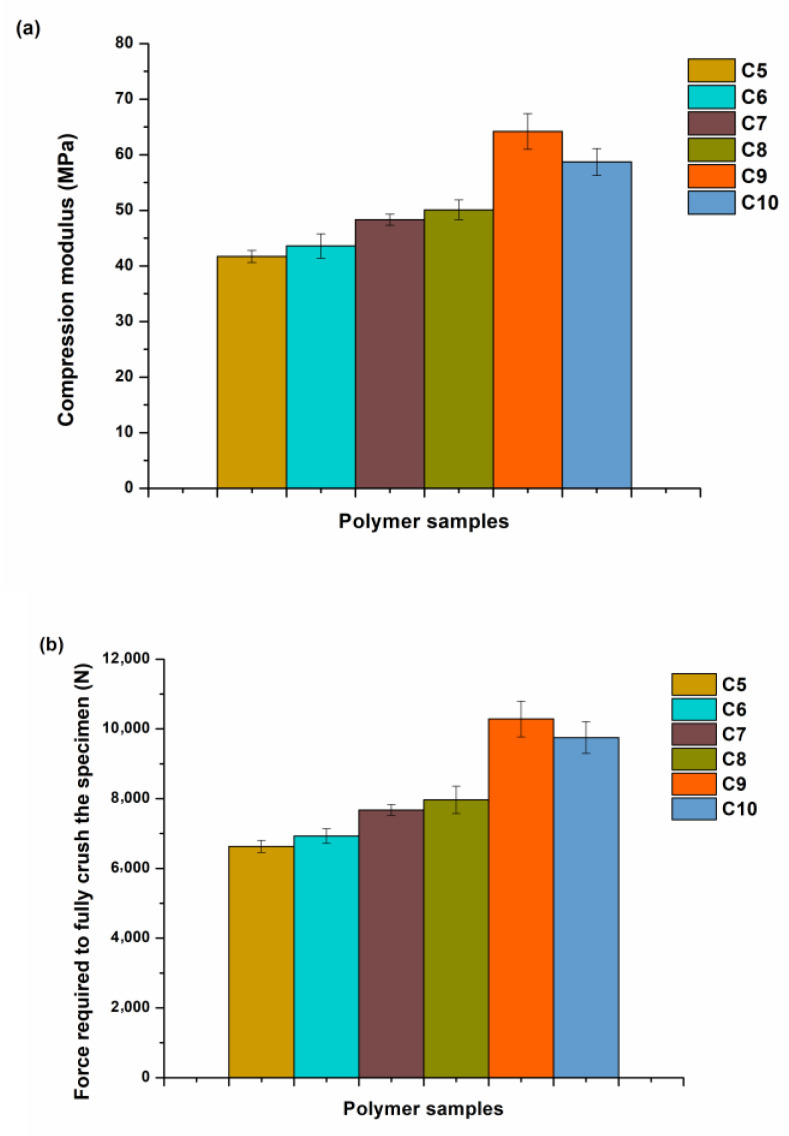
Compression characteristics of photopolymers **C5**–**C10.** (**a**) compression modulus, (**b**) force required to fully crush the specimen.

The compression test showed similar results to the tensile test. The compression modulus and the force required to fully crush the specimen increased with the increase in the THFA content going from the polymer sample **C5** to **C9** (Figure 10). The polymer sample **C10** had lower values of the compression modulus and the force required to fully crush the specimen (58.7 MPa, 9754 N) than those of the polymer sample **C9** (64.2 MPa, 10,287 N) due to increased brittleness of the polymer **C10**.

## 4. Conclusions

A series of new biobased photopolymers have been developed using a combination of green chemistry and green engineering concepts. Photocurable resins were composed of tetrahydrofurfuryl acrylate, tridecyl methacrylate, and 1,3-benzenedithiol, molar ratio 0–10:0.5:0.03, respectively. The increase in the tetrahydrofurfuryl acrylate content in the photocurable resins resulted in a higher photocuring rate, increased rigidity, and mechanical and thermal characteristics of the resulting polymers. However, more than 9 mol of tetrahydrofurfuryl acrylate in the composition resulted in the formation of the brittle polymer. Polymer synthesized from the resin containing 9 mol of tetrahydrofurfuryl acrylate, 0.5 mol of tridecyl methacrylate, and 0.03 mol of 1,3-benzenedithiol was chosen as the best of the series with great thermal (T_dec.-15%_ = 365 °C) and mechanical (Young’s modulus = 0.26 MPa, compression modulus = 64.2 MPa) characteristics and photocuring rate (t_gel_ = 2.4 s). All synthesized polymers showed thermoresponsive shape-memory behavior that maintained their temporary shape below the glass transition temperature, and their ability to return to their permanent shape above the glass transition temperature. Taking into account the recent environmental problems, the obtained new biobased photopolymers with thermoresponsive shape-memory properties could be applied as sustainable polymers in 4D printing, soft robotics, biomedical devices, flexible electronics, etc.

## Figures and Tables

**Table 1:** Composition of resins **C0**–**C10**.

Resin	Amount of THFA, mol	Amount of C13-MA, mol	Amount of BDT, mol	Amount of TPOL, mol.%	Calculated Biorenewable Carbon Content, %
**C0**	0	0.5	0.03	3	74.9
**C1**	1	0.5	0.03	3	68.9
**C2**	2	0.5	0.03	3	66.9
**C3**	3	0.5	0.03	3	65.8
**C4**	4	0.5	0.03	3	65.1
**C5**	5	0.5	0.03	3	64.7
**C6**	6	0.5	0.03	3	64.4
**C7**	7	0.5	0.03	3	64.2
**C8**	8	0.5	0.03	3	64.0
**C9**	9	0.5	0.03	3	63.8
**C10**	10	0.5	0.03	3	63.7

**Table 2:** Rheological characteristics of resins **C0**–**C10**.

Resin	Storage Modulus, *G*′, MPa	Loss Modulus, *G*″, MPa	Loss Factor, tan *δ*	Complex Viscosity *η* *, MPa^.^s	Gel Point *t*_gel_, s	Induction Period, s	Shrinkage, %
**C0**	0.001	5.71·10^−8^	5.0·10^−5^	1.82·10^−5^	- *	-	0.0
**C1**	0.011	0.069	6.520	0.001	-	19.2	0.0
**C2**	0.167	0.291	1.740	0.535	-	4.8	0.0
**C3**	0.487	0.515	1.060	0.113	-	2.4	1.0
**C4**	0.634	0.545	0.860	0.013	9.6	2.4	2.0
**C5**	0.742	0.601	0.810	0.015	2.4	2.4	3.0
**C6**	1.106	0.800	0.724	0.022	2.4	2.4	4.0
**C7**	1.256	0.853	0.675	0.024	2.4	2.4	5.1
**C8**	1.340	0.825	0.616	0.025	2.4	2.4	6.1
**C9**	1.477	0.941	0.637	0.028	2.4	2.4	7.1
**C10**	1.915	1.148	0.600	0.036	2.4	2.4	8.1

*—not determined.

**Table 3:** Yield of insoluble fraction, swelling values, and thermal properties of photopolymers **C3**–**C10**.

Polymer	Yield of Insoluble Fraction, %	Swelling Value in Acetone α	Swelling Value in Toluene α	*T*_dec.−15%_, °C *	*T*_g_, °C **
**C3**	55	168	296	331	−29
**C4**	59	171	240	344	−28
**C5**	75	155	240	358	−28
**C6**	68	152	241	356	−27
**C7**	67	131	229	354	−23
**C8**	69	130	192	357	−21
**C9**	83	119	139	365	−19
**C10**	82	120	142	364	−20

*—determined from TGA curves, **—determined from DMTA curves.

## Data Availability

Not applicable.
